# Pimavanserin: Potential Treatment For Dementia-Related Psychosis

**DOI:** 10.14283/jpad.2018.29

**Published:** 2018-08-16

**Authors:** Jeffrey Cummings, C. Ballard, P. Tariot, R. Owen, E. Foff, J. Youakim, J. Norton, S. Stankovic

**Affiliations:** 1 Cleveland Clinic Lou Ruvo Center for Brain Health, 888 W. Bonneville Ave, Las Vegas, NV, USA; 2 University of Exeter Medical School, Exeter, UK; 3 Banner Alzheimer's Institute and University of Arizona College of Medicine, Phoenix, AZ, USA; 4 ACADIA Pharmaceuticals Inc., San Diego, CA, USA

**Keywords:** Dementia, psychosis, Alzheimer's disease, Parkinson's disease, frontotemporal dementia, dementia with Lewy bodies

## Abstract

Psychosis is common across dementia types with a prevalence of 20% to 70%. Currently, no pharmacologic treatment is approved for dementia-related psychosis. Atypical antipsychotics are frequently used to treat these disorders, despite significant safety concerns. Pimavanserin, a selective 5-HT2A inverse agonist/antagonist, was approved in the U.S. for treating hallucinations and delusions associated with Parkinson's disease psychosis (PDP). Patients in the pimavanserin group experienced a significant (p=0.001) improvement in Scale for the Assessment of Positive Symptoms - Parkinson's disease (SAPS-PD) scores vs. placebo. In a subgroup analysis of patients with cognitive impairment (MMSE score ≥21 but ≤24), the observed improvement on the SAPS-PD with pimavanserin (N=50) was also significant (p=0.002) and larger than in the overall study population without an adverse effect on cognition. In a Phase 2 study with pimavanserin in Alzheimer's disease psychosis, pimavanserin significantly (p=0.045) improved psychosis at Week 6 vs. placebo on the NPI-NH Psychosis Score (PS). In a prespecified subgroup of patients with a baseline NPI-NH PS ≥12, a substantively larger treatment effect (p=0.011) was observed vs. participants with NPI-NH PS <12. The results of these studies in cognitively impaired patients with PDP provided the scientific foundation for an ongoing study of pimavanserin for treating patients with dementia-related psychosis associated with the most common neurodegenerative disorders. The study uses a relapse-prevention design with the endpoint of time-to-relapse of psychosis to evaluate the long-term efficacy and safety of pimavanserin as a potential treatment for hallucinations and delusions of dementia-related psychosis.

**P**sychosis is a common feature of dementia and becomes more frequent with disease progression (1, 2, 3). Psychosis is common in neurodegenerative disorders such as Parkinson's disease dementia (PDD) and dementia with Lewy bodies (DLB) and often occurs concurrently with cognitive decline and other non-motor symptoms and sleep disturbances (4'^9^). Among patients with PD, psychosis occurs in up to 60% of patients over the course of their disease (10, 11), Similarly, psychosis occurs with varying prevalence across other neurodegenerative diseases including Alzheimer's disease (AD), Vascular dementia (VaD), and frontotemporal dementia (FTD) (Table 1). In most neurodegenerative dementias, neurobehavioral symptoms such as psychosis are more common among those with cognitive impairment (1, 2, 3). The presence of neuropsychiatric signs and symptoms in neurodegenerative diseases is predictive of increased caregiver burden, decreased quality of life, and earlier progression to nursing home care, severe dementia, and death (3, 12). Thus, there is a close relationship between the clinical manifestations of dementia-related psychosis (DRP) and morbidity/mortality in many neurodegenerative diseases (1, 5, 6, 7, 8).

**Table 1:** Prevalence of delusions and hallucinations in patients with dementia, Alzheimer's disease, Parkinson's disease, dementia with Lewy bodies

**Disease**	**Overall Psychosis Prevalence**	**Hallucinations**	**Delusions**
Alzheimer's Disease (31–38)	30%	11–17%	10–39%
Vascular Dementia (32, 33, 34, 35, 37, 36, 39)	15%	5–14%	14–27%
Dementia with Lewy Body (35, 40, 41, 42, 43)	75%	55–78%	40–57%
Parkinson–s Disease Dementia (35, 38, 40)	50%	32–63%	28–50%
Frontotemporal Dementia (44, 45)	10%	1.2–13%	2.3–6%

No pharmacological agents are approved for treating patients with DRP, and antipsychotic (AP) drugs are often prescribed off-label for treating psychosis despite safety concerns with use of these medications in this population (13). Meta-analyses of randomized, controlled trials of APs demonstrate limited efficacy for treating DRP (14, 15). The effect size for treatment is modest (effect size=0.2) for psychosis in patients with AD (16, 17, 18). Results from the Clinical Antipsychotic Trials on Intervention Effectiveness-Alzheimer's disease (CATIEAD) study showed a significant decline in cognitive function with AP use (19), and a meta-analysis of AP in dementia patients found a similar negative effect on cognitive function (20). Further, use of APs for treating patients with dementia and PD is associated with a higher risk of mortality compared with placebo (14, 20, 21, 22, 23) as well as an increased risk of morbidity (24). Hence, there is a major unmet need for pharmacological treatment of DRP that effectively manages symptoms of psychosis without compromising cognition and with an acceptable safety and tolerability profile.

Pimavanserin, a selective 5-hydroxytryptamine (HT)2A receptor inverse agonist/antagonist, has minimal affinity for dopaminergic, muscarinic, histaminergic or adrenergic receptors (25). Pimavanserin was developed on the basis of the observation that antagonism of the 5-HT2A receptor is the common feature of most approved and efficacious APs (26).

Pimavanserin is the only drug approved in the United States for treatment of hallucinations and delusions associated with Parkinson's disease psychosis (PDP) (27). Early supportive evidence of the efficacy of pimavanserin was provided from the results of two placebo-controlled clinical trials in PDP (NCT00477672; NCT00658567; data on file, ACADIA Pharmaceuticals) (28). These studies together with a pivotal Phase 3 study (29) formed the basis of approval of pimavanserin by the US Food and Drug Administration (FDA) in 2016 for the treatment of hallucinations and delusions associated with PDP.

Recently, pimavanserin was shown to improve hallucinations and delusions in patients with AD psychosis (ADP) (30). Analyses from this study also demonstrated that pimavanserin did not negatively impact cognitive function in these patients (Table 2).

**Table 2:** Completed or ongoing analyses from randomized, placebo-controlled studies with pimavanserin for neuropsychiatric disorders

**Reference**	**Study Population**	**Treatments**	**Primary Endpoint**	**Primary Outcome**
ACP-103-020 (29)	Parkinson's disease psychosis	Pimavanserin 34 mg vs. placebo	SAPS-PD change from baseline to Week 6	Significant improvement with pimavanserin vs. placebo
ACP-103-020 (46)	Parkinson's disease psychosis	Pimavanserin 34 mg vs. placebo	SAPS-PD at Week 6 stratified by baseline MMSE	Significant improvement in both groups, but more robust in cognitively impaired
ACP-103-019 (30)	Alzheimer's disease psychosis	Pimavanserin 34 mg vs. placebo	NPI-NH psychosis at Week 6	Significant improvement for pimavanserin vs. placebo
ACP-103-019 (47)	Alzheimer's disease psychosis	Pimavanserin 34 mg vs. placebo	NPI-NH psychosis score at Week 6 by severity	Significant and more robust response in the severe subgroup
NCT03325556 [ACP-103-045]	Dementia-related psychosis	Pimavanserin (20 mg and 34 mg flexible dosing) vs. placebo	Time from randomization to relapse; Time from randomization to all-cause discontinuation	Ongoing

The findings of efficacy for pimavanserin in the PDP and ADP populations indicate that pimavanserin may have a favorable treatment effect on psychotic features across many neurodegenerative dementing illnesses. Here we review the pimavanserin clinical development program leading to the approval for PDP along with the data from the study in ADP leading to a proposed trial in DRP across a spectrum of neurodegenerative diseases. The rationale and methodology for DRP development is discussed.

## A phase 3 study of Pimavanserin for Pakinson's disease psychosis

The efficacy of pimavanserin in the treatment of hallucinations and delusions associated with PDP was demonstrated in a Phase 3, double-blind, randomized, placebo-controlled study (29).

Patients satisfying diagnostic criteria for PDP were randomized to pimavanserin 34 mg or placebo for a 6-week treatment period. The study included a 2-week screening, baseline (Day 1), 6 weeks of treatment, and a follow up visit 4 weeks after study drug discontinuation. During the 2-week screening period, patients received brief psychosocial therapy (45). The primary efficacy endpoint was mean change from baseline to Week 6 in the SAPS-PD score.

This study demonstrated clinically and statistically significant superiority of pimavanserin 34 mg over placebo in treatment of hallucinations and delusions in patients with PDP. A 5.79 point improvement (least square (LS) mean change) at Week 6 was observed with pimavanserin compared to a 2.73 point improvement for placebo in the SAPS-PD score. This represents a clinically meaningful change with a treatment difference of 3.06 points (p=0.001; effect size 0.50). The effect size of 0.50 indicates a robust effect compared with the 0.2 effect size typically reported with APs (16, 17, 18). In addition, pimavanserin was generally well tolerated with no effects on motor function as measured by the Unified Parkinson's Disease Rating Scale (UPDRS) Parts II+III.

## Subgroup analysis of outcome by baseline MMSE

Patients with dementia (Mini-Mental State Examination (MMSE) score <20) were excluded in the pivotal clinical trial (30); but some patients exhibited a limited degree of cognitive impairment. A post hoc subgroup analysis conducted from the Phase 3 study, evaluated randomized patients according to the presence or absence of cognitive impairment, defined as a MMSE score of 21 to 24 for cognitive impairment versus ≥25 for non-impaired (46). The cognitively impaired subgroup constituted about 25% of the overall study population (pimavanserin, n=29; placebo, n=21). The primary endpoint of this analysis was mean change from baseline to Week 6 for the SAPS-PD score.

**Figure 1:**
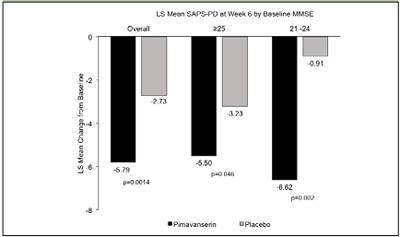
LS mean change in the SAPS-PD score to Week 6 for pimavanserin and placebo in the overall population and by baseline MMSE score (46)

Patients with cognitive impairment (MMSE score 21–24) demonstrated a 6.62 point improvement (LS mean change) in the SAPS-PD score with pimavanserin at Week 6 compared to a 0.91 point improvement with placebo, representing a treatment difference of 5.71 points (p=0.002) (Figure 1). The observed effect size (Cohen's *d*) in the subgroup of patients with PDP and dementia was 0.99. This compares to a treatment difference of 3.06 in the overall study population. In the non-cognitively impaired group (MMSE score ≥25) the LS mean change from baseline to Week 6 for the SAPS-PD was −5.50 with pimavanserin vs. −3.23 with placebo with a treatment difference of 2.27 (p=0.046). At Week 6, among the cognitively impaired subgroup for Clinical Global Impressions-Improvement (CGI-I) score, the mean difference from baseline for pimavanserin vs. placebo was 1.0 (p=0.012), and for the non-impaired, the mean difference for pimavanserin vs. placebo was −0.6 (p=0.022).

The results from this subgroup analysis suggest that pimavanserin is efficacious in PDP patients with cognitive impairment and may exhibit a more robust effect in this subgroup of patients. No notable differences were observed for the incidence of adverse events between impaired and non-impaired groups.

## A Phase 2 study of Pimavanserin for ALzheimer's disease psychosis

A completed study suggests that pimavanserin is effective in reducing hallucinations and delusions in patients with ADP (30).

This was a Phase 2, 12-week, randomized, double-blind, placebo-controlled, single-center study to assess the safety and efficacy of pimavanserin 34 mg once daily in nursing home residents with ADP (30). The pre-specified primary and secondary endpoints were evaluated at Week 6 of treatment. Eligible patients were required to have a score ≥4 on either the hallucinations or delusions component or a combined hallucinations and delusions score of ≥6 on the Neuropsychiatric Inventory-Nursing Home Version (NPI-NH). During the screening period, patients received brief psychosocial therapy. The primary efficacy endpoint was change from baseline to Week 6 for the NPI-NH psychosis score (delusions + hallucinations domains).

A total of 181 patients were randomized (n=90 pimavanserin and n=91 placebo) with 178 patients were included in the full analysis set (n=87 pimavanserin and n=91 placebo). The mean age of patients was 85.9 years. The mean baseline NPI-NH psychosis score for all patients was 9.8 with comparable mean scores in the pimavanserin (9.5) and placebo (10.0) groups. The mean baseline MMSE score for all patients was 10.1.

For the primary endpoint — drug-placebo difference on change from baseline in NPI-NH sychosis score at Week 6 — pimavanserin demonstrated a significant (p=0.045) treatment effect vs. placebo with a treatment difference of −1.84 and a Cohen's *d* effect size of 0.32. Response on the NPI-NH (defined as ≥30% improvement from baseline to Week 6) was observed in 55.2% of subjects in the pimavanserin group and 37.4% of subjects in the placebo group (p=0.016); ≥50% improvement occurred in 50.6% of subjects in the pimavanserin group and 34.1% of subjects in the placebo group (p=0.024). Mean changes from baseline for the MMSE score and UPDRS Part III (motor function) scores were minimal and comparable for pimavanserin and placebo. Patients were followed until week 12. Both pimavanserin and placebo-treated patients continued to improve. The drug-placebo difference at Week 12 — a secondary endpoint — did not reach statistical significance.

This study suggests that pimavanserin may be effective in treating hallucinations and delusions in patients with ADP. Pimavanserin had no adverse effects on motor function (UPDRS) or cognition (MMSE).

## Subgroup analysis of patients with more severe psychosis at baseline

**Figure 2:**
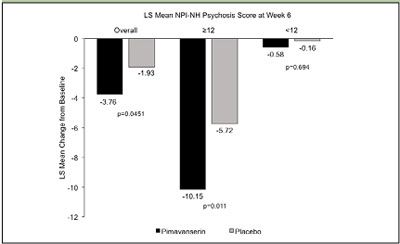
LS mean change from baseline to Week 6 for the NPI-NH psychosis score among the overall population from a randomized, placebo-controlled study (30) and in subgroups by severity of psychosis (47)

In the analytic plan of the ADP Phase 2 study data, a pre-specified subgroup analysis was conducted in patients who had more severe psychotic symptoms (hallucinations and delusions) at baseline as measured by NPI-NH psychosis score (30, 47). This pre-specified analysis corroborated the primary endpoint results and showed that patients with more severe psychotic symptoms at baseline (NPI-NH psychosis score ≥12) experienced greater improvement compared to patients with less severe symptoms at baseline (NPI-NH psychosis score <12) (Figure 2). In patients with baseline NPI-NH psychosis score ≥12, LS mean change to Week 6 was −10.15 with pimavanserin vs. −5.72 with placebo (delta= 4.43, Cohen's *d* = 0.734, p=0.011), which was a substantively larger treatment effect compared to patients with NPI-NH psychosis score <12 (delta= 0.42, Cohen's *d* = 0.077).

**Figure 3:**
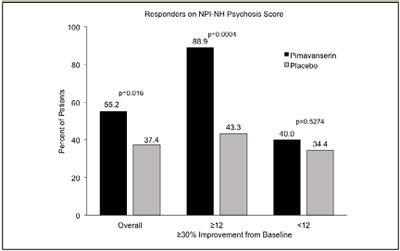
Response rate at Week 6 for the NPI-NH psychosis score among the overall population from a randomized, placebo-controlled study (30) and in subgroups by severity of psychosis (47)

Prespecified responder analyses in residents with more severe baseline symptoms also demonstrated the significant effect of pimavanserin compared with placebo in patients with ADP (Figure 3). A significantly greater proportion of the pimavanserin patients showed ≥30% improvement from baseline and ≥50% improvement from baseline on their NPI-NH psychosis score. Among patients with a NPI-NH psychosis score ≥12, response for pimavanserin and placebo (defined by ≥30% improvement from baseline to Week 6) was observed in 88.9% vs. 43.3% (p<0.001) and, when defined by .50% improvement was 77.8% vs. 43.3% (p=0.008), respectively.

Thus, in the subgroup of patients with more severe psychotic symptoms at baseline, significant improvements in mean NPI-NH psychosis score and in NPI-NH responder rates were observed with pimavanserin vs. placebo. These findings were consistent with the observations in the overall population and demonstrate the robust significant treatment effect of pimavanserin vs. placebo in patients with severe symptoms of psychosis at baseline.

## Pimavanserin for the treatment of DRP

The efficacy and safety of pimavanserin for treatment of psychotic symptoms in dementia are being evaluated in an ongoing study: a Double-blind, Placebo-controlled, Relapse Prevention Study of Pimavanserin for the Treatment of Hallucinations and Delusions Associated With Dementia Related Psychosis (Clinicaltrials.gov. NCT03325556). The study is designed to evaluate the efficacy of pimavanserin in preventing relapse of psychotic symptoms in patients with DRP following 12 weeks of open-label treatment with pimavanserin followed by blinded randomized withdrawal of treatment or continued pimavanserin therapy.

Eligible patients will include those who meet criteria for all-cause Dementia according to National Institute on Aging-Alzheimer's Association (NIA-AA) guidelines (48) as well as satisfying clinical criteria for one of the following disorders (with or without cerebrovascular disease): PDD (49), DLB (50), possible or probable AD, frontotemporal degeneration spectrum disorders (51, 52, 53) or vascular dementia (54). In addition, patients will have an MMSE score ≥6 and ≦24, have psychotic symptoms for at least 2 months, SAPS H+D ≥10; CGI-S ≥4 (moderately ill), and have at least one SAPS H+D global item ≥4 (corresponding to moderate or severe psychosis). For those patients taking a cholinesterase inhibitor and/or memantine, the dose of this medication must remain stable for at least 12 weeks prior to baseline. Patients on AP medications at screening will need to be tapered off their medication prior to baseline, if medically appropriate, or they will be excluded. Brief psychosocial therapy will be administered during screening to identify patients who respond to non-pharmacological therapy, and thus who are no longer appropriate for enrollment.

After a 4-week screening period, approximately 360 patients will enter an open-label period with flexible dosing of pimavanserin. At 12 weeks, patients who met specific criteria for sustained response to open-label treatment at both 8 and 12 weeks will be randomized to a daily dose of pimavanserin 20 or 34 mg (based on their open-label dose) or placebo for 26 weeks. The primary outcome is time from randomization to relapse of psychosis in the double-blind period. Relapse is defined as a ≥30% increase from Week 12 on the SAPS-H+D Total Score and a CGI-I score of 6 or 7; treatment with an additional antipsychotic for DRP; patient withdrawing from the study for lack of efficacy; or hospitalization for worsening DRP. A key secondary outcome is time from randomization to “all-cause” discontinuation from the double-blind period.

There are several advantages of the relapse prevention design of this study. First, it maximizes the duration of exposure to a potentially effective treatment (pimavanserin) and minimizes the duration of exposure to placebo (55, 56, 57). A second advantage is the enrichment of the study population with an open-label, run-in phase, which helps to minimize inclusion of non-responders. Also, Brief Psychosocial Therapy will be used at screening to eliminate patients who respond to non-pharmacological therapies, ensuring that most patients in need, receive pharmacological treatment. In addition, the withdrawal design affords the advantage of offering potentially therapeutic medication to all participants at enrollment, making it more feasible to enroll persons with active psychosis who might otherwise be unwilling or unable to consider trial participation. This addresses a major barrier to enrollment in trials when treatment is perceived as most necessary. Similar designs have been used successfully with a number of antipsychotics and antidepressants to demonstrate long-term efficacy and safety in a range of psychiatric indications (57). This design is aligned with 2017 American Psychiatric Association guidelines for use of antipsychotic drugs in dementia patients: if a drug demonstrates no efficacy after 4–6 weeks, therapy should be discontinued. If a drug demonstrates efficacy within 16 weeks, an attempt should be made to taper off medication to determine if ongoing therapy is necessary.

The conceptual basis for using pimavanserin in DRP is based on the observation that a common feature of antipsychotics is antagonism of the 5-HT2A receptor (26) and that this effect is applicable regardless of the associated neuropathology (plaques, tangle, Lewy bodies, TDP-43, vascular lesions). The emergence of psychotic symptoms in many types of dementia suggests that diverse pathologies may give rise to a common symptom complex; this final pathway may be subject to modification from 5-HT2A receptor antagonism.

## Summary

Clinical evidence is now available that supports potential efficacy of pimavanserin in DRP. This includes results from a Phase 3 study in patients with PDP (29), a secondary analysis of 25% of patients enrolled in this study who also had cognitive impairment (MMSE of 21 to 24) where the observed effect size (Cohen's *d*) in the subgroup of patients with PDP and cognitive impairment was 0.99, and a Phase 2 study in patients with ADP (30) indicating a robust effect in patients with more severe psychosis (47).

Across two different models of DRP (PD and AD) pimavanserin has demonstrated meaningful efficacy larger than that reported with current off-label treatments. These clinical data, coupled with a substantial body of research, suggest that psychotic symptoms can manifest independent of the underlying dementia subtype.

In summary, based on the overlap in clinical presentation and pathology, as well as in management of psychotic symptoms in patients with dementia, and importantly, the positive clinical trial results in two neurodegenerative patient populations (PD and ADP), pimavanserin's effect in patients experiencing hallucinations and delusions associated with DRP across a number of neurodegenerative disorders is being investigated.

*Acknowmedgement:* The authors acknowledge the editorial assistance of Richard S. Perry, PharmD in the preparation of this manuscript, which was supported by ACADIA Pharmaceuticals Inc., San Diego, CA.

*Funding:* This study was funded by ACADIA Pharmaceuticals Inc., San Diego, California. All authors as well as the sponsor were involved in the design and conduct of the study; the collection, analysis, and interpretation of data; in the preparation of the manuscript; and in the review or approval of the manuscript.

*Conflicts of interest:* JLC has provided consultation to ACADIA, Accera, Actinogen, ADAMAS, Alkahest, Allergan, Alzheon, Avanir, Axovant, Axsome, BiOasis Technologies, Biogen, Boehinger-Ingelheim, Eisai, Genentech, Grifols, Kyowa, Lilly, Lundbeck, Merck, Nutricia, Otsuka, QR Pharma, Resverlogix, Roche, Samus, Servier, Suven, Takeda, Toyoma, and United Neuroscience companies. Dr. Cummings is supported by Keep Memory Alive (KMA), COBRE grant # P20GM109025; TRC-PAD # R01AG053798; DIAGNOSE CTE # U01NS093334. PT reports the following (pertinent for the last two years): consulting fees from Abbott Laboratories, AbbVie, AC Immune, Acadia Pharmaceuticals, Auspex, Boehringer-Ingelheim, Chase Pharmaceuticals, Eisai, Glia Cure, Insys Therapeutics, and Pfizer; Consulting fees and research support from AstraZeneca, Avanir, Eli Lilly, Lundbeck, and Roche; Research support only from Amgen, Avid, Biogen, Elan, Functional Neuromodulation (f(nm)), GE Healthcare, Genentech, Novartis, Targacept, NIA, and Arizona Department of Health Services; he is a contributor to a patent owned by the University of Rochester, “Biomarkers of Alzheimer's disease” and owns stock options in Adamas, and he has received research support, consulting fees, and serves on an advisory board for Merck and Co. Dr. Ballard has received grants and personal fees from ACADIA and Lundbeck, personal fees from Heptares, Roche, Lilly, Otsuka, Orion, GlaxoSmithKline, and Pfizer. JY, EF, SS, RO, and JN are employees of and stockholders in ACADIA Pharmaceuticals Inc.

*Ethical standard:* The study adheres to the Declaration of Helsinki human protection guidelines and was reviewed by ethical standards boards for all participating sites.
